# Maternal and Foetal Cardiovascular Effects of the Anaesthetic Alfaxalone in 2-Hydroxypropyl-**β**-cyclodextrin in the Pregnant Ewe

**DOI:** 10.1155/2013/189843

**Published:** 2013-10-24

**Authors:** Anna Andaluz, Laura Santos, Félix García, Rosa I. Ferrer, Laura Fresno, Xavier Moll

**Affiliations:** Department of Animal Medicine and Surgery, Faculty of Veterinary Medicine, Universitat Autonoma de Barcelona (UAB), Bellaterra, 08193 Barcelona, Spain

## Abstract

The objective of this study was to determine the pharmacodynamics effects of the anaesthetic alfaxalone in 2-hydroxypropyl-**β**-cyclodextrin in pregnant sheep after the intravenous injection of a 2 mg/kg weight dose. Six pregnant Ripollesa sheep, weighing 47.1 ± 4.4 kg, were used. Twenty-four hours after instrumentation, sheep were anaesthetized with intravenous alfaxalone in cyclodextrin. Time to standing from anaesthesia was 30.0 ± 10.81 min. Foetal heart rate increased significantly during the first 5 min after alfaxalone administration. Significant differences were observed in maternal diastolic arterial blood pressure between minute 10 and minutes 90, 120, 150, 180, 210, and 240. Significant differences were observed for foetal systolic arterial blood pressure between 5 and 30 min after alfaxalone administration. Significant differences in foetal pH were detected during the entire study period, whereas maternal pH returned to baseline values by 60 min after alfaxalone administration. The present study indicated that alfaxalone in 2-hydroxypropyl-**β**-cyclodextrin administered as an intravenous bolus at 2 mg/kg body weight produced minimal adverse effects and an uneventful recovery from anaesthesia in pregnant sheep and their foetus.

## 1. Introduction

General anaesthesia is often indicated for surgery during pregnancy or for caesarean section. Nevertheless, drugs given to the mother during pregnancy or for caesarean section can affect the ability of the neonate to survive in and adapt to its new environment. Neonatal drug elimination is usually slow, and many drugs have prolonged effects in the neonatal period [1]. Use of a short-acting hypnotic providing rapid loss of consciousness with minimal cardiovascular and respiratory suppression for both mother and foetus and rapid recovery is necessary for these patients.

Pregnant ewes have been popularly used as animal models for placental transfer research as well as for the determination of the anaesthetic effects in the foetus. Although extrapolation of results from animals to humans should be done with caution because of anatomical and physiological differences, sheep have been shown to be the most appropriate experimental model for studies of placental transfer of drugs, especially the soluble ones, such as general anaesthetics [2, 3]. It has been shown that halothane, isoflurane, and sevoflurane may produce hypotension both in the mother and the foetus [4–8]. They can also decrease the foetal (halothane, isoflurane, and sevoflurane) and maternal (isoflurane) heart rate and cause an increase in *P*aCO_2_ in the mother and the foetus, inducing a foetal acidosis [4, 8]. Injectable anaesthetics have also been evaluated. Propofol, in addition to causing hypotension in the mother, causes an increase in heart rate of the foetus and an impaired acid-base balance, in both mother and foetus, with significant foetal acidosis [9]. Recent studies on etomidate have shown that, although it causes slight alterations in acid-base equilibrium of foetuses, it has very little effect on blood pressure and heart rate making it a good candidate for use in pregnant patients. Nevertheless, etomidate administration has been associated with significant adrenal suppression, limiting its use in pregnant patients [10, 11].

Alfaxalone (3-alpha-hydroxy-5-alpha-pregnane-11,20-dione) in 2-hydroxypropyl-*β*-cyclodextrin (HPCD) (Alfaxan, Jurox Pty. Ltd.) is the new formulation of this neurosteroid anaesthetic characterized by not producing histamine release. In veterinary medicine, it is used to induce and maintain general anaesthesia [12–15]. Following intravenous injection, it has a rapid onset of action, rapid redistribution, and a short terminal half-life [16]. Experimental studies investigating the cardiorespiratory and anaesthetic effects of alfaxalone in dogs [12, 17, 18], cats [14, 19, 20], rabbits [21], and sheep [22] have shown minimal cardiorespiratory depression, making alfaxalone an acceptable induction agent. Moreover, alfaxalone administration has not been associated with adrenal suppression [23], so it could be useful in pregnant patients. Nevertheless, to our knowledge, no published studies have been conducted on the effects of this drug formulation during gestation.

The aim of the present study was to determine the cardiovascular effects and the changes in acid-base balance both for ewes that were 4 months pregnant and their foetuses after the administration of a single IV bolus of alfaxalone. 

## 2. Material and Methods

All procedures were approved by the Ethical Commission of Animal and Human Experimentation (Spanish Government) under the auspices of the Ethical Commission of the Universitat Autònoma de Barcelona (Authorization nos. DARP 4544 and CEEAH 791).

Six pregnant Ripollesa breed sheep weighing (mean ± SD) 47.1 ± 4.4 kg were included in the study. Mean gestational age was 120 ± 3.8 days (term 147–150 days).

### 2.1. Animal Preparation

The anaesthetic and surgical techniques used in our study were similar to those described by Andaluz et al. [9] with some minor modifications. Sheep were premedicated with 0.02 mg/kg buprenorphine IM (Buprex, Schering-Plough Laboratories) and 0.2 mg/kg meloxicam IM (Metacam, Boehringer Ingelheim). A single dose of 4 mg/kg propofol (Propofol-Lipuro 1%, Braun) was administered for anaesthesia induction through an 18-gauge polyurethane catheter (Vasocan, Braun) placed in the right cephalic vein. The trachea was intubated with a 9–10 mm endotracheal tube, and anaesthesia was maintained with 2–2.5% isoflurane (IsoVet, B. Braun) in 100% oxygen through a circle breathing system. Ventilation was controlled using an intermittent positive pressure ventilator (SAV 2500, B. Braun) in order to maintain normocapnia during the entire anaesthetic period. An orogastric tube was placed and maintained while the ewe was anaesthetized to prevent regurgitation and aspiration pneumonia. All animals received an infusion of lactated Ringer's solution at a rate of 10 mL/kg/h during the perioperative period and IV antibiotic therapy with 20 mg/kg cephazolin (Kurgan, Normon Laboratories) was administered via the cephalic vein.

Each sheep was positioned in dorsal recumbency. The neck and the abdomen were prepared aseptically. Firstly, a small incision was made in the skin over the neck. The carotid artery was dissected for a 32 cm, 14G plain polyurethane catheter (Cavafix Certo, B. Braun) placement. The maternal carotid artery catheter was used for blood gases determination and for heart rate and blood pressure measurement. Then, a midline laparotomy was performed, and through a small hysterotomy, the foetus was delivered partially to permit adequate exposure for insertion of a 71 cm, 14G plain polyurethane catheter (Drucafix-Splittocan, B. Braun) into the carotid artery. This catheter was used for blood gas determination and for heart rate and foetal blood pressure measurement. The skin of the foetus was incised over the carotid artery. Fine dissection of the vessels and placement of the catheter were performed and the catheter was immediately heparinised and the blood pressure was measured to ensure the functionality of the catheter. The skin incision was sutured and the foetus returned to the uterus. Care was taken during surgery to minimize loss of amniotic fluid. The placenta and the uterus were closed as previously described [9]. The foetal catheter was tunnelled subcutaneously through the ewe's flank, exteriorized, and stored in a plastic pouch sewn on the skin of the flank. The laparotomy incision was closed in a routine manner.

Throughout the surgical process, temperature, cardiac frequency, respiratory frequency, capnography, arterial pressure, pulse, and electrocardiography were monitored using a multifunction patient VetCare monitor (B. Braun).

Following recovery from anaesthesia, sheep were returned to their pen and allowed to recover for 24 h before the experimental phase began. During this period, ewe and foetal arterial catheters were heparinised every 8 h to avoid occlusion. Animals were treated with SC buprenorphine (0.02 mg/kg every 6 h) and meloxicam (0.1 mg/kg every 24 hours) for pain control after surgery. 

### 2.2. Experimental Design

During the study period, maternal and foetal heart rate, arterial blood pressure (systolic, diastolic, and mean), and maternal respiratory rate were determined at different times using a multifunction patient VetCare monitor (B. Braun). Foetal and maternal arterial blood samples were also taken at different times for acid-base variables evaluation using an i-STAT Portable Clinical Analyzer. Blood samples for acid-base determination were collected into a 1 mL heparinised insulin syringe and processed within 5 min. 

All efforts were made to keep the sheep in sternal recumbency during the anaesthetic periods. The appearance of adverse effects (salivation, regurgitation, myoclonus, and apnoea) was recorded if present. Time to recovery from anaesthesia (standing) was also recorded.

Before starting the study, a control period of 60 min was established. Baseline values for heart rate, blood pressure, respiratory rate, and *Et*CO_2_ represent the means of four determinations taken at 15 min intervals, while baseline values for blood gases were measured only once at minute 30.

After the 60 min control period, a single IV bolus of 2 mg/kg alfaxalone was administered through the cephalic vein catheter and both maternal and foetal cardiovascular variables were measured at 2, 5, 10, 15, 20, 30, 45, 60, 90, 120, 150, 180, 210, and 240 min after alfaxalone injection. Blood samples for acid-base status evaluation were taken simultaneously from mother and foetus at 5, 15, 30, 60, 120, and 240 min after alfaxalone injection.

All animals were monitored and received a lactated Ringer's solution therapy at 60 mL/kg/day for the entire study period. If episodes of apnoea occurred, artificial ventilation was initiated until spontaneous respiration was regained.

Data were analyzed using a statistical computer software program (SPSS v19.0, SPSS Ibérica). Normality was tested using the Shapiro-Wilk statistic. The results were analyzed by analysis of variance using a mixed model, including time as fixed effect and sheep as the random effect. When significant differences were found, a Bonferroni post hoc analysis for pairwise comparisons was performed. A value of *P* < 0.05 was considered to be significant. To evaluate time of anaesthesia and time to standing, one-sample *t*-test was used.

## 3. Results

Time to standing from anaesthesia, after a single IV bolus of 2 mg/kg body weight of alfaxalone in ewes, was 30.0 ± 10.81 min (mean ± SD). No adverse effects were observed after alfaxalone injection. 

### 3.1. Maternal Variables

Maternal cardiovascular and acid-base variables are shown in Tables 1 and 2.

No significant differences were observed in maternal heart rate (HR) and systolic (SAP) and mean (MAP) arterial blood pressure during the entire study period. Significant differences were observed in diastolic arterial blood pressure (DAP) between minute 10 and the minutes 90, 120, 150, 180, 210, and 240. 

A significant decrease from baseline values was observed in maternal pH 5 minutes after alfaxalone administration (*P* < 0.05); this returned to baseline at 30 min. This alteration was accompanied by a significant increase in *P*aCO_2_ 5 min after alfaxalone administration from the baseline value and a significant decrease in *P*aO_2_ during min 5 and 15 compared with the entire study period (*P* < 0.05). 

### 3.2. Foetal Variables

Foetal cardiovascular and acid-base variables are shown in Tables 3 and 4.

Foetal heart rate was significantly increased during minutes 2 and 5 after alfaxalone administration (*P* < 0.05). Foetal arterial blood pressure remained constant during the entire study period compared to the control period. Only significant differences in SAP between minutes 5 and 30 were observed.

Foetal pH decreased and *P*aCO_2_ increased following a similar pattern as that described for maternal pH although significant differences (*P* < 0.05) from the baseline value remained during the entire study period only for pH. No significant differences were observed for *P*aCO_2_ and *P*aO_2_.

## 4. Discussion

To the authors' knowledge, this is the first published study in which alfaxalone in HPCD has been evaluated in pregnant ewes.

The alfaxalone dosages used and the times chosen for recording all the variables were those described previously for alfaxalone in nonpregnant sheep by Andaluz et al. [22]. 

Time to standing was longer in the present study (30.0 ± 10.81 min) than that described in nonpregnant sheep (22.0 ± 10.6 min) [22]. As pregnancy-associated alterations in physiological function affect the uptake, distribution, and disposition of anaesthetic agents [24], the results observed in the present study may indicate that elimination of alfaxalone in pregnant sheep could be slower than in nonpregnant ones. Several investigations have postulated that the dose of drugs in pregnant patients should be decreased by 25% compared to the usual dose [24]. This reduction has not been performed in this study, so that this could have prolonged the recovery time from anaesthesia. 

As described for nonpregnant sheep, apnoea or other adverse effects did not occur in any of the animals of this study. However, apnoea is one of the most frequently reported side effects after alfaxalone use. The slow administration rate, as postulated by Andaluz et al. [22] in nonpregnant ewes, or the possibility that sheep are less sensitive to the apnoea produced by this drug could explain these results. Although apnoea was not observed in any of the sheep, it seems that alfaxalone tends to produce a respiratory depression that is manifested by the transient decrease in pH and the increase in *P*aCO_2_. However, pH remained within the clinically acceptable range for sheep (7.48–7.58) [25] throughout the study, so it is not expected that those alterations would have an important clinical implication. This respiratory depression is also seen in foetuses, in which the pH is decreased significantly during the entire study period. These results agree with those reported for alfaxalone in nonpregnant sheep [22] and are very similar to those described by Fresno et al. [11] for etomidate administration in ewes. This may show that both, alfaxalone and etomidate, present a good stability on the respiratory system, which makes them optimal for anaesthesia induction in pregnant patients. The results also resemble those described for sevoflurane and isoflurane in pregnant ewes [8], especially when they are administered at 1.5 to 2 minimal alveolar concentration (MAC). 

Moreover, as observed in nonpregnant sheep [22], alfaxalone did not produce significant cardiovascular depression in the ewe. The increase in heart rate observed in nonpregnant sheep, attributed to the lateral recumbent position of the animals during the study, has not been observed in pregnant sheep. The cardiovascular adverse effects observed in the previous study may have been avoided by the maintenance of sheep in sternal recumbency in the present study. These results show that the cardiovascular safety of alfaxalone in pregnant sheep is similar to that described for etomidate [11], isoflurane, and sevoflurane [8] and higher than that described for propofol, as the latter produces a marked hypotension immediately after its administration in pregnant ewes [9]. However, cardiovascular stability of etomidate appears to be superior to that of alfaxalone in the foetus. Intravenous bolus of alfaxalone, as observed previously with propofol [9], produces a significant increase in heart rate. This effect has been associated with foetal response to maternal stress during anaesthesia induction and with foetal distress caused by a reduction in the uterine blood flow [26]. The decrease in foetal *Pa*O_2_ may also have contributed to the increase in heart rate. Oxygen supplementation prior to alfaxalone administration as well as during early recovery may have diminished this effect. For this reason, although sheep used in the present study did not receive oxygen supplementation, the authors strongly recommend to oxygenate patients before and during induction of anaesthesia.

Although inhalant anaesthetic agents may be used for anaesthesia induction, this technique is not the most recommended in many patients as it can be slow, delaying the time of endotracheal intubation and increasing the probability of vomiting and aspiration pneumonia. Moreover, induction with inhalant anaesthetic agents may cause agitation when no sedative agents have been used, which often happens with pregnant patients. Because etomidate may result in the suppression of foetal serum cortisol concentrations [27] and propofol in severe hypotension, especially in patients with haemodynamic instability, alfaxalone in HPCD may be a useful option for anaesthesia induction in pregnant animal patients if care is taken to ensure optimal oxygenation and ventilatory support.

It can be concluded that the administration of a single bolus of alfaxalone in HPCD has no apparent major adverse effects on the pregnant ewe when compared to those described in nonpregnant animals or those described in pregnant ewes undergoing inhalant or propofol anaesthesia. However, as respiratory effects on the foetuses could be important, the authors recommend to provide 100% oxygen to ewes in order to prevent hypoxia and foetal respiratory acidosis during alfaxalone anaesthesia. 

## Figures and Tables

**Table 1:** Changes in maternal cardiovascular variables following administration of a single intravenous bolus of 2 mg/kg of alfaxalone in a 2-hydroxypropyl-*β*-cyclodextrin formulation (mean ± SD).

	HR (bpm)	SAP (mm Hg)	DAP (mm Hg)	MAP (mm Hg)	RR (rpm)
Baseline values	126.00 ± 20.56	96.00 ± 19.06	63.16 ± 14.71	760.50 ± 16.18	48.83 ± 15.98
2 min	130.60 ± 16.86	81.16 ± 16.00	57.16 ± 40.60	67.00 ± 39.02	30.00 ± 8.29
5 min	133.40 ± 18.80	90.00 ± 19.66	65.5 ± 19.59	73.16 ± 16.43	29.33 ± 13.54
10 min	134.20 ± 23.49	103.3 ± 25.15	80.83 ± 14.16	88.67 ± 19.02	37.00 ± 14.41
15 min	134.50 ± 28.72	102.7 ± 14.45	79.00 ± 14.41	86.83 ± 13.01	39.67 ± 14.56
20 min	140.20 ± 24.63	101.50 ± 10.23	78.67 ± 11.47	87.5 ± 10.03	39.67 ± 8.98
30 min	150.30 ± 24.48	93.5 ± 17.63	64.00 ± 10.90	73.83 ± 9.84	42.67 ± 11.22
45 min	133.20 ± 12.92	96.33 ± 18.18	64.17 ± 20.72	78.00 ± 19.24	47.33 ± 9.26
60 min	130.80 ± 18.73	91.8 ± 13.54	64.00 ± 7.24	74.00 ± 10.61	57.00 ± 25.73
90 min	115.00 ± 13.37	89.8 ± 19.76	53.80 ± 15.15*	63.00 ± 13.32	62.40 ± 26.32
120 min	117.75 ± 25.95	81.6 ± 8.23	50.60 ± 17.99*	63.8 ± 14.41	71.20 ± 36.15
150 min	125.20 ± 12.02	93.25 ± 17.85	56.00 ± 25.17*	71.75 ± 20.18	60.80 ± 41.12
180 min	120.80 ± 12.81	80.2 ± 10.47	59.8 ± 15.31*	68.4 ± 12.58	57.60 ± 36.50
210 min	121.60 ± 15.37	91.6 ± 12.22	61.20 ± 22.55*	74.80 ± 17.22	48.00 ± 14.42
240 min	109.50 ± 12.71	90.80 ± 24.78	62.00 ± 32.83*	76.00 ± 27.91	50.80 ± 20.95

*Significant differences from min 10.

HR: heart rate; SAP: systolic arterial pressure; DAP: diastolic arterial pressure; MAP: mean arterial pressure; RR: respiratory rate.

**Table 2:** Changes in maternal acid-base variables following administration of a single intravenous bolus of 2 mg/kg of alfaxalone in a 2-hydroxypropyl-*β*-cyclodextrin formulation (mean ± SD).

	pH	PaCO_2_ (mm Hg)	PaO_2_ (mm Hg)	HCO_3_^−^ (mmol/L)	Base excess (mmol/L)
Baseline values	7.57 ± 0.04	27.33 ± 2.92	89.67 ± 15.15^†,‡^	24.82 ± 2.26	2.67 ± 2.34
5 min	7.50 ± 0.06*	33.72 ± 1.59*	68.50 ± 16.16	25.52 ± 2.94	2.17 ± 3.76
15 min	7.49 ± 0.04*	32.33 ± 2.94	70.67 ± 16.44	25.45 ± 2.83	3.00 ± 3.95
30 min	7.54 ± 0.05	29.32 ± 3.09	83.83 ± 18.05^†,‡^	25.00 ± 4.50	2.50 ± 5.24
60 min	7.52 ± 0.06	28.82 ± 0.91	90.20 ± 4.97^†,‡^	23.50 ± 2.62	0.80 ± 3.63
120 min	7.53 ± 0.05	29.56 ± 3.28	99.40 ± 10.74^†,‡^	24.68 ± 3.88	2.00 ± 4.47
240 min	7.51 ± 0.06	29.72 ± 1.98	103.00 ± 11.25^†,‡^	24.06 ± 4.03	1.00 ± 5.15

*Significant differences from baseline values.

^†^Significant differences from min 5.

^‡^Significant differences from min 15.

**Table 3:** Changes in foetal cardiovascular variables following maternal administration of a single intravenous bolus of 2 mg/kg of alfaxalone in a 2-hydroxypropyl-*β*-cyclodextrin formulation (mean ± SD).

	HR (bpm)	SAP (mm Hg)	DAP (mm Hg)	MAP (mm Hg)
Baseline values	197.16 ± 34.80	59.66 ± 8.09	36.66 ± 3.72	46.16 ± 4.62
2 min	251.00 ± 77.31*	66.83 ± 8.63	48.16 ± 9.80	54.16 ± 7.02
5 min	271.50 ± 33.94*	73.33 ± 18.26	47.00 ± 9.12	57.16 ± 8.32
10 min	217.00 ± 39.35	59.00 ± 8.60	44.17 ± 7.96	50.5 ± 7.42
15 min	222.2 ± 34.58	59.67 ± 10.52	38.17 ± 8.20	47.83 ± 6.52
20 min	208.50 ± 32.26	56.67 ± 12.53	40.33 ± 5.57	46.00 ± 7.61
30 min	208.20 ± 32.25	53.00 ± 7.07^†^	38.50 ± 5.50	46.50 ± 4.03
45 min	198.70 ± 27.69	61.33 ± 14.05	40.33 ± 7.60	49.00 ± 8.92
60 min	187.50 ± 28.70	56.83 ± 10.09	40.83 ± 5.26	48.00 ± 6.87
90 min	193.33 ± 22.23	59.83 ± 9.74	43.5 ± 6.47	50.16 ± 5.49
120 min	226.83 ± 45.77	65.83 ± 17.26	45.66 ± 13.30	56.00 ± 14.68
150 min	189.17 ± 28.80	59.66 ± 5.81	45.66 ± 7.84	52.66 ± 5.42
180 min	191.5 ± 27.60	64.66 ± 7.44	47.83 ± 7.75	54.83 ± 7.41
210 min	209.00 ± 34.89	69.66 ± 15.05	48.00 ± 9.57	56.16 ± 13.89
240 min	187.50 ± 22.74	62.33 ± 7.00	46.33 ± 10.09	53.16 ± 5.84

*Significant differences from baseline values.

^†^Significant differences from min 5.

HR: heart rate; SAP: systolic arterial pressure; DAP: diastolic arterial pressure; MAP: mean arterial pressure; RR: respiratory rate.

**Table 4:** Changes in foetal acid-base variables following maternal administration of a single intravenous bolus of 2 mg/kg of alfaxalone in a 2-hydroxypropyl-*β*-cyclodextrin formulation (mean ± SD).

	pH	PaCO_2_ (mm Hg)	PaO_2_ (mm Hg)	HCO_3_^−^ (mmol/L)	Base excess (mmol/L)
Baseline values	7.47 ± 0.02	37.30 ± 2.45	20.00 ± 9.34	27.17 ± 2.32	3.50 ± 2.35
5 min	7.39 ± 0.05*	43.12 ± 4.26	16.33 ± 7.81	26.25 ± 2.81	1.00 ± 2.76
15 min	7.43 ± 0.04*	41.00 ± 4.57	21.17 ± 7.70	26.85 ± 4.18	2.33 ± 4.41
30 min	7.42 ± 0.03*	39.65 ± 4.34	20.00 ± 7.72	25.70 ± 3.21	1.17 ± 3.54
60 min	7.44 ± 0.03*	38.83 ± 3.03	19.33 ± 9.27	26.53 ± 2.36	2.33 ± 2.73
120 min	7.41 ± 0.04*	40.39 ± 1.74	17.49 ± 8.61	25.62 ± 2.36	0.96 ± 3.06
240 min	7.39 ± 0.05*	41.51 ± 1.78	19.20 ± 8.25	25.86 ± 3.10	1.35 ± 2.98

*Significant differences from baseline values.
